# Buffalo Medical and Surgical Association

**Published:** 1890-07

**Authors:** W. H. Bergtold

**Affiliations:** Secretary


### BUFFALO MEDICAL AND SURGICAL ASSOCIATION.

Reported by W. H. BERGTOLD, M. D., *Secretary*.

Regular monthly meeting, held June 3d, at 8.45 P. M., in the parlors of Hotel Iroquois.

The president, Dr. A. A. Hubbell, in the chair.

In the absence of the secretary, Dr. Jacob Falk acted in that capacity.

Dr. T. Haven Ross was proposed for membership.

Dr. Floyd S. Crego then read a paper on Psycho-Therapeutics or Treatment by Hypnotism and Suggestion.

### DISCUSSION.

Dr. Putnam said the same experiments quoted by the speaker were reported years before our present time, and these tricks have been traditional for years. Patients are easily hypnotized after severe operations have been performed on them. Cases seen the first time are more difficult to bring under the influence of the operator. In

France, Dr. Putnam was able to hypnotize many cases, but on his return home he did not find it so easy as his experience in Europe. Some authors claim that the condition is altogether one of suggestion. Hypnotism as a treatment will be limited in this country. Here the condition will be an entirely new thing, and the cases will not lend the coöperation that is needful. One case of insomnia that Dr. Putnam had, failed entirely to be hypnotized. He has cured nocturnal enuresis in children by suggestion.

Dr. Hopkins said that mental suggestion is the only scientific fact of homeopathy; whatever the result may be, it is suggestion. In bringing the patient to a condition of hypnotism, coöperation is the word, not concentration.

Dr. Howe thinks there is no coöperation necessary on the part of the patient in many subjects. Cases seen by him at Charcot's clinique, in Paris, were readily hypnotized, though the hesitation of the party to readily submit may cause delay in the success of the attempt.

Dr. Ring thought the question will ere long be recognized by the legislature.

Dr. Van Peyma asked for information regarding hypnotism in animals.

Dr. Hopkins here stated that sometimes in sending patients to eminent men of the profession in other cities, they would return to him immensely improved, with a memory of every little thing which this doctor did for them during the consultation, and drew attention to this as a species of coöperation.

Dr. Putnam believes that hypnotism frequently produces diseases. A certain proportion of hysterical cases will be made worse. Prof. Meynert, of Vienna, prefers to heal disease, not give cause for it.

